# Ozone Chemically Enhanced Backwash for Ceramic Membrane Fouling Control in Cyanobacteria-Laden Water

**DOI:** 10.3390/membranes10090213

**Published:** 2020-08-30

**Authors:** Stéphane Venne, Onita D. Basu, Benoit Barbeau

**Affiliations:** 1 Department of Civil and Environmental Engineering, Carleton University, 1125 Colonel By Drive, Ottawa, ON K1S 5B6, Canada; stephane.venne31@gmail.com; 2 Department of Civil, Geological and Mining Engineering, Polytechnique, C.P. 6079 Succursale Centre-Ville, Montréal, QC H3C 3A7, Canada; benoit.barbeau@polymtl.ca

**Keywords:** ceramic ultrafiltration, cyanobacteria fouling, ozone, chemical cleaning, chemically enhanced backwash

## Abstract

Membrane fouling in surface waters impacted by cyanobacteria is currently poorly controlled and results in high operating costs. A chemically enhanced backwash (CEB) is one possible strategy to mitigate cyanobacteria fouling. This research investigates the potential of using an ozone CEB to control the fouling caused by Microcystis aeruginosa in filtered surface water on a ceramic ultrafiltration membrane. Batch ozonation tests and dead-end, continuous flow experiments were conducted with ozone doses between 0 and 19 mg O3/mg carbon. In all tests, the ozone was shown to react more rapidly with the filtered surface water foulants than with cyanobacteria. In addition, the ozone CEB demonstrated an improved mitigation of irreversible fouling over 2 cycles versus a single CEB cycle; indicating that the ozone CEB functioned better as the cake layer developed. Ozone likely weakens the compressible cake layer formed by cyanobacteria on the membrane surface during filtration, which then becomes more hydraulically reversible. In fact, the ozone CEB reduced the fouling resistance by 35% more than the hydraulic backwash when the cake was more compressed.

## 1. Introduction

The increasing frequency and intensity of harmful cyanobacteria (algae) blooms in surface freshwater, due primarily to eutrophication and climate change [1], poses challenges to drinking water treatment plants. The rapid changes in cell densities and their tendency to float can result in inefficient treatment by coagulation and cell breakthrough to settled water [2]. Meanwhile, low-pressure ultrafiltration (UF) membranes can almost completely remove cyanobacteria cells, including the smallest ones (Microcystis aeruginosa, 3–6 μm), from water by size exclusion [3] and with minimal cell breakage [4]. However, membrane productivity rapidly decreases due to fouling by cyanobacteria cells and natural organic matter (NOM) present in surface water. Cyanobacteria cells form a compressible cake layer on the membrane [4,5] whereas NOM adsorbs on the membrane surface as well as within the membrane pores [6,7]. Consequently, fouling increases operation costs and remains the greatest obstacle to the application of membranes in drinking water treatment, especially the hydraulically irreversible fouling fraction (i.e., fouling not removed by hydraulic backwashes) [8,9].

To remove hydraulically irreversible fouling, chemical cleaning is most commonly executed as a clean-in-place (CIP) operation, which requires that the membrane be soaked in chemicals for extended periods of time [9]. The drawbacks of a CIP are that it requires process adjustments, high chemical concentrations, and results in costly downtime [9]. A chemically enhanced backwash (CEB) is an alternative strategy in which a chemical, at a relatively lower concentration compared to a CIP, is combined with a hydraulic backwash for an in-situ cleaning process that results in downtimes similar to those of hydraulic backwashes [10].

Ozone is of particular interest for CEB applications, with ceramic membranes, as it reacts rapidly with organic material in water [11] and only small exposures are required to damage cyanobacterial cells [2]. Furthermore, the catalytic decomposition of ozone by ceramic materials leads to the formation of stronger and less selective oxidants, making the combination of ozone and ceramic membranes very attractive and yielding new opportunities for membrane fouling control [11]. In comparison, polymeric membranes are damaged by repeated exposure with oxidants [8], although polymeric membranes with improved ozone resistance are being developed.

The efficiency in which ozone can clean ceramic membranes has been previously demonstrated in the literature. For instance, when ozone was used in a CIP procedure to clean a ceramic ultrafiltration membrane fouled by humic acids and alginate fractions of NOM, over 98% of the unified membrane fouling index (UMFI) was recovered within an hour [12]. In that application, a dissolved ozone solution was recirculated with a normalized dose of 0.5 mg O3 per mg of organic carbon on the membrane at the start of the CIP. The reported performance of the ozone CIP was comparable to a 4-h CIP with a mixed solution of sodium hydroxide and hypochlorite.

Sartor et al. [13] used continuous in-situ ozonation, a technique in which ozone is continuously dosed in the membrane module during filtration, to control ceramic membrane fouling by surface water. In this study, the rate of fouling was reduced and hydraulic backwashes maintained the membrane’s specific flux at approximately 80% of its original value, which is up to 4 times higher than without in-situ ozonation. In the presence of the cyanobacterium M. aeruginosa (2 × 106 cells/mL, DOC: 1.67 mg/L), Wei et al. [14] also reported that in-situ ozonation (0-5 mg/L) reduced the rate of UF membrane fouling. The authors of both studies hypothesized that the reactions between ozone, the extrapolymeric substances, and the natural organic matter in the feed water lead to the formation of a more porous cake layer on the membrane surface. This more porous cake resulted in a membrane foulant resistance that was smaller than foulant resistance caused by the cake formed without in-situ ozonation.

The application of in-situ ozonation reduces the rate of membrane fouling but this type of application is energy-intensive and requires the continuous addition of ozone to be effective [15]. A greater ozone demand is expected as ozone can react with all the feed components, not only what fouls the membrane. Furthermore, in treatment applications involving cyanobacteria, ozone can induce the release of the cells’ internal organic metabolites [2] which often exacerbates hydraulically irreversible fouling [14]. Furthermore, these metabolites can be toxic [1], and permeate through the membrane [14] causing concerns for human health. In these cases, there is an increase in the permeate’s dissolved organic carbon concentration and an adsorptive media such as granular activated carbon is required downstream to remove these components and ensure regulatory compliance [13].

Chemically enhanced backwashing (CEB) does not suffer from the disadvantages of CIPs and continuous in-situ ozonation. However, research addressing the mechanisms and kinetics involved in CEB fouling control applications is limited. The impact of an ozone CEB on membrane fouling was quantified, in which ozone could almost completely remove the fouling of a ceramic microfiltration treating municipal wastewater [16]. The authors suggested that this performance was due to increased ozone reactions with foulants present within the membrane pores. Most of the other studies regarding CEB applications used a chlorine CEB for membrane fouling control in seawater desalination and feed water that has a low particulate organic matter concentration. The CEB was rarely the primary focus of these studies, which generally address the application of low-pressure membranes as a pretreatment step to reverse osmosis. Additionally, the frequency of the CEB and the oxidant dose varied greatly between studies (between every 1 to 24 h; 1 to 500 mg/L Cl2), and the chosen doses where not justified [10]. Studies analyzing the impact of CEB parameters on performance have not been found for surface water applications nor cyanobacteria-laden water applications.

The objective of this research is to demonstrate the potential of ozone chemically enhanced backwashes to control the fouling of ceramic ultrafiltration membranes by cyanobacteria in a filtered surface water and to understand the cleaning mechanisms involved in the process. This will be achieved by: (1) comparing the performance of an ozone CEB to the performance of a hydraulic (water-only) backwash, (2) determining the rate of ozone demand and organic foulant removal from the membrane during an ozone CEB, and (3) evaluating the effect of ozone dose on foulant removal and membrane resistance. This research will help determine the importance of different CEB parameters in order to optimize the impact of an ozone chemically enhanced backwash on membrane fouling control.

## 2. Materials and Methods

### 2.1. Cyanobacterial Culture

*Microcystis aeruginosa* (CCPC 633, non-toxic strain) was purchased from the Canadian Phycological Culture Centre (Waterloo, Ontario, Canada). It was incubated in 3N-BBM growth media, at 21 ± 2 °C, with constant aeration, and under a 12-h light/dark cycle to simulate normal growth conditions (2000 lux, Phillips). *M. aeruginosa* was chosen for this study as it is the predominant freshwater cyanobacterial species [1] and has the smallest single cell diameter. Small single cells are problematic as they are the most likely to not be removed by coagulation and sedimentation [3]. The culture’s growth was monitored by direct cell enumeration using an improved Neubauer hemocytometer (Marienfield). On average, the single cells had a diameter of 4.11 ± 0.75 μm once in the early stationary phase, between 30 to 36 days after inoculation.

### 2.2. Feed Water Characteristics

To fully understand the impact of different foulants present in surface water impacted by cyanobacteria on the performance of a chemically enhanced backwash, three feed solutions were used in this study: surface water (SW), cyanobacteria-spiked ultrapure water (Cyano-UW), and cyanobacteria-spiked surface water (Cyano-SW). The surface water was obtained from the Sainte-Rose Drinking Water Treatment Plant, of which the intake is located in the Rivière des Mille-Îles (Laval, Québec, Canada). The surface water used in the experiments was collected after alum coagulation and flocculation, sedimentation, and sand-anthracite filtration to minimize fluctuations in water quality throughout experiments.

To prepare the Cyano-UW feed solution, cyanobacteria cells were spiked into ultrapure water (Milli-Q™). Cyanobacteria cells were harvested in the early stationary growth phase and were separated from the growth medium by centrifugation at 10,000 *g* and 4°C for 15 min [4,14]. To obtain a cyanobacteria cell concentration of 5 × 10^5^ cells/mL in the feed solution, a concentration that could represent the fraction of a large bloom that breakthrough conventional treatment [17], the centrifuged volume of growth medium was determined based on the growth medium’s cell concentration.

The preparation of the Cyano-SW feed was similar to the preparation of the Cyano-UW feed except that the cyanobacteria cells were spiked directly into the filtered surface water sampled at the Sainte-Rose treatment plant instead of ultrapure water.

The parameters of the three feed solutions are listed in Table 1. Since divalent ions have been shown to impact membrane fouling [18], the total hardness of all three feed solutions was adjusted to 60 mg CaCO_3_/L using calcium chloride dihydrate (Fisher Scientific). During the experiments, the temperature of the feeds was maintained at room temperature (23 ± 1 °C).

### 2.3. Bench-Scale Membrane Filtration System

**Figure 1:**
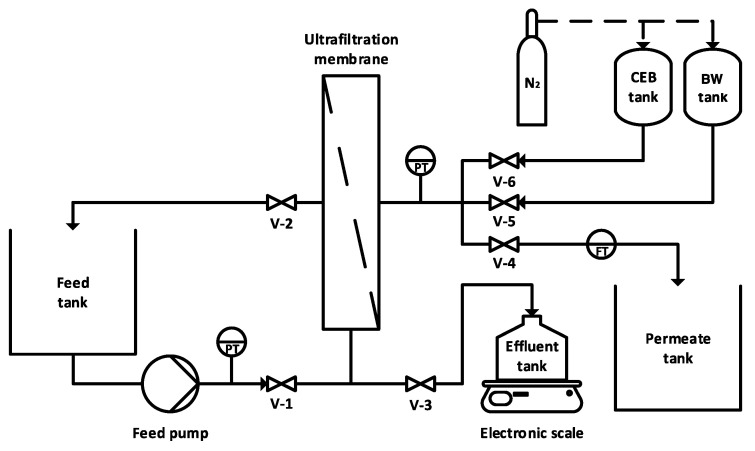
Schematic diagram of bench-scale ultrafiltration membrane system (PT: pressure transducer, FT: flow meter). Valves 1 and 4 are open during dead-end filtration; valves 3 and 5 are open during hydraulic backwashes; valves 3 and 6 are open during chemically enhanced backwashes; and valves 1 and 2 are open during the end-of-experiment chemical washes.

Experiments were conducted with a ceramic ultrafiltration membrane (Atech Innovations, Germany) installed on a semi-automated bench-scale filtration system. A schematic representation of the system is presented in Figure 1. The membrane was tubular, with a surface area of 95 cm^2^ and a molecular weight cut-off of 150 kDa. The membrane surface was composed of zirconium dioxide (ZrO_2_) and supported by an α-aluminum oxide (α-Al_2_O_3_) layer.

The membrane was operated in dead-end and was fed by a gear pump (drive: Ismatec BVP-Z; head: MicroPump L3468) to obtain a constant permeate flux of 200 LMH. Given the high mechanical strength of ceramic membranes, the permeate flux was set higher than average to accelerate fouling. The permeate flowrate (flow sensor: McMillan Flow 101-3T) and trans-membrane pressure (transducers: Omega PX309-100G5V) were measured every 30 s. The trans-membrane pressure was calculated by subtracting the pressure measured at the membrane outlet (PT ahead of V-4) from the feed pressure (PT ahead of V-1, see Figure 1). Hydraulic (water-only) backwashes were initiated every 30 min and had a duration of 27 s (equivalent to 3 membrane volume replacements). Ultrapure water at room temperature and pressurized to 0.78 atm (11.5 psig) by nitrogen gas was used to obtain a flowrate of approximately 600 LMH during the hydraulic backwash.

At the end of each experiment, the membrane was washed with a mixed solution of sodium hydroxide and sodium hypochlorite (pH 12, 500 mg/L as Cl_2_, 35 °C) [12]. To do so, the feed tank in Figure 1 was replaced with the cleaning solution, which was recirculated at a cross-flow velocity of 0.1 m/s for 1 h and then left to soak for 3 h. This was followed by a hydrochloric acid wash to remove any residual organic and inorganic foulants. The HCl solution (pH 2) was recirculated at 0.1 m/s for 1 h, left to soak for 2 h, and recirculated again for 1 h (adapted from [19]). Afterwards, the membrane system was flushed with ultrapure water until the pH of the permeate was stable.

### 2.4. Experimental Plan

Three sets of experiments were conducted to address the research objectives: batch ozonation tests, baseline membrane performance tests, and ozone CEB tests.

#### 2.4.1. Batch Ozonation Tests

The purpose of the batch ozonation tests is to determine and compare the reactivity of ozone with the different foulants in the feed solutions, without the interference of the CEB’s hydraulic force or the membrane material, which reacts with ozone. To do so, a liter of each feed solution was placed in 2-L borosilicate glass beakers, spiked with ozone, and continuously stirred. A small volume of highly concentrated ozone stock solution (50–60 mg O_3_/L) was added to obtain a normalized ozone dose of 2 milligrams per milligram of TOC in the feed solution. The greatest sample dilution factor caused by the addition of ozone stock was 18% and was observed in the Cyano-SW feed, which had the largest TOC content as seen in Table 1. The stock was prepared by bubbling gaseous ozone, which was produced by a bench-top ozone generator (Ozone Solutions TG-10), in ultrapure water at 4 °C. To minimize degassing, the aliquot of ozone stock solution was dosed with a syringe, feed solutions were covered with floating polytetrafluoroethylene lids, and water samples for analysis were taken with a syringe. Solutions were left to react with ozone for 30 min. Experiments were conducted at room temperature (23 °C). The ozone concentration in solution throughout the tests was determined using the indigo colorimetric method described elsewhere [20].

#### 2.4.2. Baseline Membrane Performance Tests

The purpose of the baseline tests is to determine the membrane fouling mechanisms involved when filtering the feed solutions and to evaluate their impact on fouling control by hydraulic backwashes. To do so, the feed solutions were filtered on the bench-scale membrane system discussed in Section 2.3. The control condition consisted in hydraulic backwashes using a solution without ozone for the same duration/frequency as a 2 mg O_3_/mg C CEB. The performance of these extended backwashes will be compared to the performance of CEBs in the ozone CEB tests.

#### 2.4.3. Ozone CEB Tests

Two series of CEB experiments were conducted. Firstly, 30-min long ozone chemically enhanced backwashes were initiated after 2 h of filtration on the bench-scale membrane system described in Section 2.3, during which the residual ozone concentration in the effluent and the cumulative organic matter removed from the membrane were monitored. This experiment was repeated with each feed solution. A control experiment, which is a 30-min CEB of the clean membrane, was also conducted to estimate the ozone demand from the ceramic membrane itself given its reactivity with ozone. Overall, the purpose of the 30-min CEB is to determine the foulant removal kinetics by ozone and to compare them to the kinetics observed during the batch ozonation tests.

Secondly, the purpose of the second set of ozone CEB experiments is to evaluate the impact of the ozone dose on the post-CEB membrane resistance. To do so, the Cyano-SW feed was filtered for 6 h on the bench-scale membrane system described in Section 2.3, during which an ozone CEB was initiated every 2 h. The experiment was repeated with different CEB ozone doses, which were varied by adjusting the CEB duration. To normalize the results, the doses were expressed as a function of the organic carbon mass remaining on the membrane surface prior to the CEB (m_CEB_), as determined by mass balances. The mass balance calculation is expressed in Equations (1a) and (1b), where TOC_f_, TOC_p_, and TOC_BW_ are the TOC concentrations in the feed, permeate, and cumulative hydraulic backwash effluent, respectively; whereas V_f_, V_p_, V_BW_, and V_m_ are the volumes of feed filtered, the volume of permeate (which is equal to the volume of feed filtered since the membrane is operated in dead-end filtration), the cumulative backwash effluent, and the internal membrane volume, respectively.

(1a) $$m_{CEB}=m_{f}-m_{p}-\sum m_{BW}$$

(1b) $$m_{CEB}=\left(TOC_{f}*V_{f}\right)-\left(TOC_{p}*V_{p}\right)-\left(TOC_{BW}*V_{BW}-TOC_{f}*V_{m}\right)$$

In both sets of experiments, each CEB was preceded by a hydraulic backwash to specifically evaluate the impact of ozone on the hydraulically irreversible fouling fraction. This strategy also mimics the usual CEB approach which is applied after a regular BW. The ozonated water used in the CEBs was prepared by diffusing gaseous ozone directly into the CEB reservoir filled with ultrapure water at 4 °C, similarly to the method presented in Section 2.4.1. Once the ozone concentration reached 40 mg/L, the reservoir was pressurized to 0.78 atm (11.5 psig) and the CEB was started immediately. The dissolved ozone concentration in the stock CEB solution and in the CEB effluent was determined with the indigo method [20]. To minimize ozone degassing when sampling, the extremity of the CEB effluent line was submerged in an overflowing 45 mL vial. Samples could then be taken from the center of the vial with a syringe.

### 2.5. Analytical Methods

In addition to the specific monitored parameters discussed above, the pH, the turbidity (Hach 2100), the total and dissolved organic carbon concentrations (Sievers M5310C On-Line TOC Analyzer), the ultraviolet absorbance at 254 nm (Cary UV-vis, Varian) were measured. Fluorescence excitation-emission matrices (FEEM) were also obtained (Shimadzu 5301PC). Excitation and emission wavelengths were set to range between 220 and 600 nm, with an excitation increment of 10 nm, an excitation slit width of 10 nm, an emission slit width of 5 nm, and a sampling interval of 1.0 nm. In the batch tests, these measurements were taken for the feed before and after ozonation. In the baseline and CEB experiments, the measurements were taken for the feed, permeate, hydraulic backwash (cumulative for all hydraulic backwash between two CEBs), and CEB effluents.

## 3. Results and Discussion

### 3.1. Ozone Kinetics in Batch Tests

**Figure 2:**
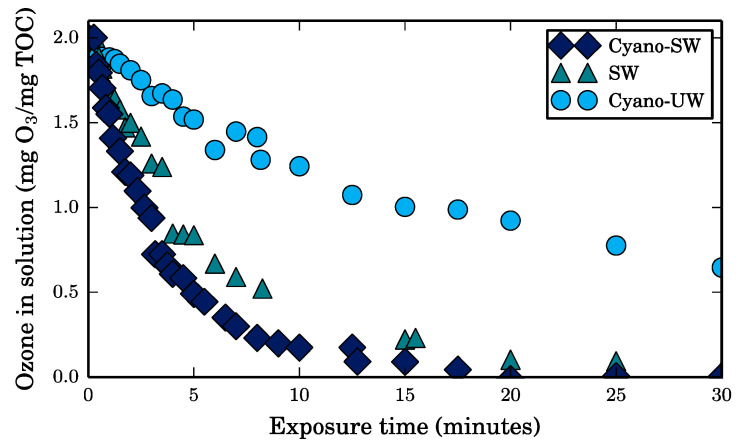
Ozone mass in solution (normalized) in 1 L batch solutions of surface water (SW), cyanobacteria-spiked ultrapure water (Cyano-UW), and cyanobacteria-spiked surface water (Cyano-SW). Ozone spiked to obtain a concentration of 2 mg of ozone per mg of total organic carbon (TOC) in solution. Experiments conducted at 23 °C.

In the batch test experiments, each feed was spiked with two milligrams of ozone per milligram of TOC in solution and continuously stirred. The ozone mass in solution was measured over time. As illustrated in Figure 2, ozone reacts more rapidly with the components in surface water (SW) than with the cyanobacteria cells (Cyano-UW).

However, as the TOC concentrations measured before and after ozonation are indeed almost identical (SW: 2.71 to 2.64 mg/L, Cyano-UW: 2.19 to 2.13 mg/L, Cyano-SW: 4.86 to 4.64 mg/L), it can be concluded that there was insignificant mineralization of both the surface water natural organic matter and the cyanobacteria cells. As the structure and chemical properties of the various organic molecules that the TOC measurement quantifies change when the molecules are oxidized, their reactivity with ozone also changes [11]. The increases in DOC to TOC ratios (SW: 0.00% increase since all DOC initially, Cyano-UW: 70% increase, Cyano-SW: 31%) and the decreases in UV_254_ absorbances (SW: 69%, Cyano-UW: 46%, Cyano-SW: 58%) after ozonation supports this statement. Moreover, in the Cyano-UW experiment, approximately 30% of the ozone dose was still in solution after 30 min and the incomplete transformation of TOC to DOC suggests that residual particulate organic matter, mainly comprised of cyanobacteria cell debris, reacted slowly with ozone.

### 3.2. Baseline Membrane Fouling and Hydraulic Reversibility

**Figure 3:**
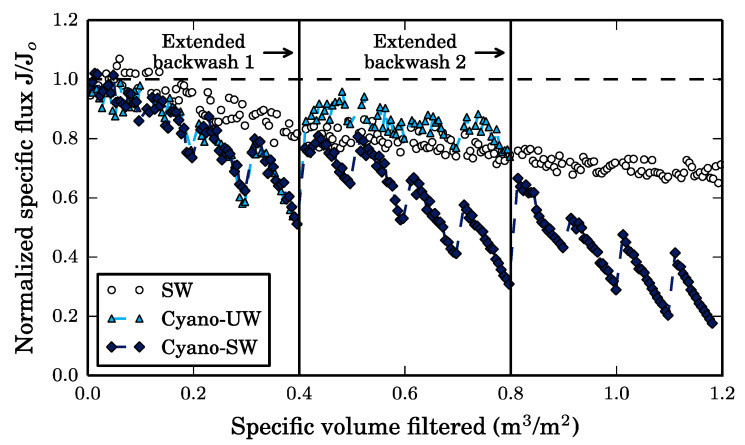
Decrease in the membrane’s specific flux during constant flux, dead-end filtration (200 LMH) of different feed solutions. The horizontal dashed line indicates the clean membrane flux. The Cyano-UW data is almost fully superimposed by the Cyano-SW data. The Cyano-UW data does not extend passed 0.4 m^3^/m^2^, which represents 2 h of filtration, for fear that cyanobacteria cells would start to significantly deteriorate in the unbuffered ultrapure water.

Ultrafiltration reduced the turbidity, TOC, DOC, and UV_254_ of the SW feed solution by 41%, 41% (all TOC is DOC), 6.8%, and 12%, respectively. In contrast, the turbidity, TOC, DOC, and UV_254_ were reduced by 96%, 99%, 84% and 100% during the filtration of Cyano-UW feed and were reduced by 97%, 43%, 4.9% and 47% during the filtration of Cyano-SW feed. Since the membrane is operated in dead-end filtration, the solution components removed by the membrane are therefore, the membrane foulants. As a result, the membrane specific flux (*J*), which is defined as the permeate flux (200 LMH) divided by the trans-membrane pressure, decreases with increasing volumes of feed filtered, as illustrated in Figure 3.

The decrease in specific flux caused by the SW and Cyano-UW solutions indicate that both surface water components and cyanobacteria cells foul the membrane. However, since the specific flux decrease caused by the Cyano-SW is almost identical to the decrease caused by Cyano-UW feed solution, it can be concluded that the cyanobacteria cells have a greater impact on total fouling than surface water components.

**Figure 4:**
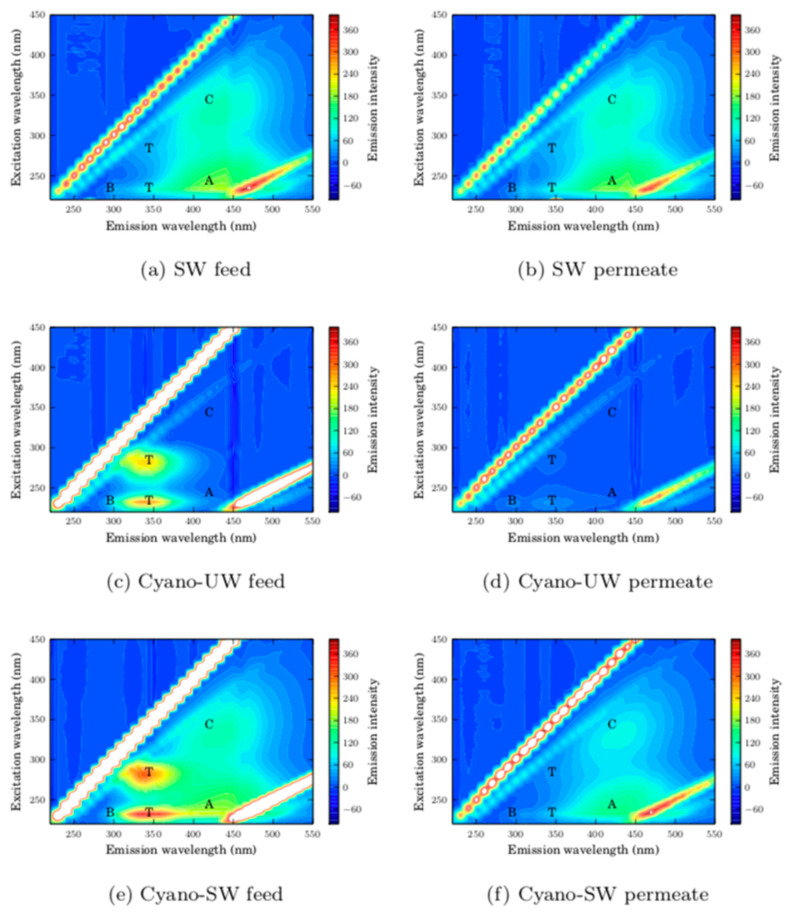
Fluorescence excitation-emission matrices of membrane feed and permeate streams. The peaks A (excitation/emission: 237-260/400-500 nm) and C (excitation/emission: 300-370/400-500 nm) represent the humic-like and fulvic-like substances. The B (excitation/emission: 225-237/309-321 and 275/310 nm) and T (excitation/emission: 225-237/340-381 and 275/340 nm) peaks represent the tyrosine protein-like and the tryptophan protein-like substances [21].

As seen in the fluorescence excitation-emission matrices (FEEMs) of the feed and membrane permeate solutions presented in Figure 4, the surface water organic matter was mainly composed of humic-like and fulvic-like substances (peaks A and C), as well as a small fraction of polysaccharides (peak T). The FEEM of the SW membrane permeate is similar to the FEEM of the SW feed solution, which indicates that the majority of the organic matter in SW is not intercepted by the membrane. This is also reflected by the small DOC removal of 6.8%. It is likely that the majority of NOM molecules are smaller than the membrane pores given that the water was collected post-sand/anthracite filtration at the drinking water treatment plant. Consequently, it is expected that the removed surface water NOM is adsorbed on the membrane surface and within its pores. This is supported by the decreasing specific membrane flux for SW (Figure 3), which appears to reach a steady-state, as it would in an adsorption isotherm prior to exhaustion.

In comparison, the presence of cyanobacteria cells in ultrapure water (Cyano-UW) is identified by two peaks in the proteinic regions of the FEEM (T peaks). In this case, the FEEM of the Cyano-UW permeate indicates that the cells are completely removed by the membrane, as also suggested by the 99.0% TOC removal. Given that the cyanobacteria cells are much larger than the membrane pores, they form a cake layer on the membrane surface. Interestingly, the intensity of the A and C FEEM peaks in the permeate are reduced when filtering the Cyano-SW. It is likely that the cyanobacteria cake acts as a filter aid.

**Figure 5:**
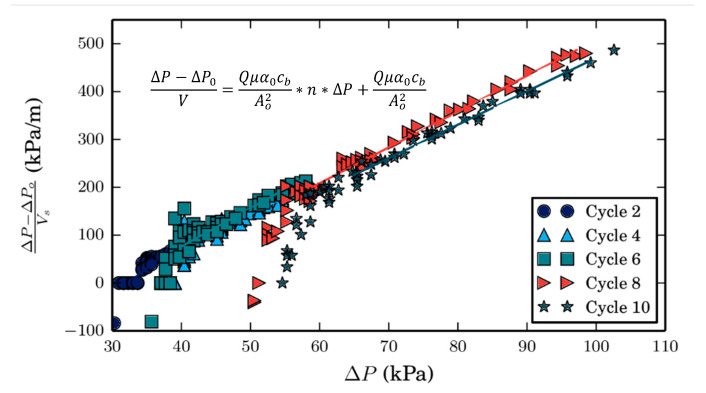
Compressibility of foulant cake layer formed on the membrane surface during the filtration of Cyano-SW. A cycle is defined as the filtration period between two hydraulic backwashes.

Moreover, the cake formed by cyanobacteria is shown to be compressible by fitting the trans-membrane pressures (ΔP) measured in the Cyano-SW baseline experiment to the model developed by Chellam and Xu [22] for microbial suspensions (Equation (2)).

(2) $$\Delta P=\Delta P_{0}+\frac{Q\mu \alpha_{0}\left(1+n\Delta P\right)c_{b}}{A_{0}^{2}}V$$

where ΔP_0_ is the trans-membrane pressure at the beginning of a filtration cycle, Q is the flow rate, A_0_ is the membrane surface area, μ is the water viscosity, $\alpha_{0}$ is the specific cake resistance at null pressure, c_b_ is the bulk concentration, V is the volume filtered, and n is the cake compressibility factor. The fitted data are presented in Figure 5.

The average compressibility factor of the cake layer is 3.15 ± 0.08 × 10^−2^ m^2^/N, which is relatively large (a value of 0 represents an incompressible cake) [22]. The compressibility factor is obtained by calculating the average of the slopes of the plots’ linear portions. All fits had an R^2^ > 0.90. Similar compressibility factors were obtained when fitting the Cyano-UW data (3.02 ± 0.11 × 10^−2^). It is believed that the non-linear portion of data is the result of under-developed cake formation, where other types of fouling mechanisms might be taking place.

**Figure 6:**
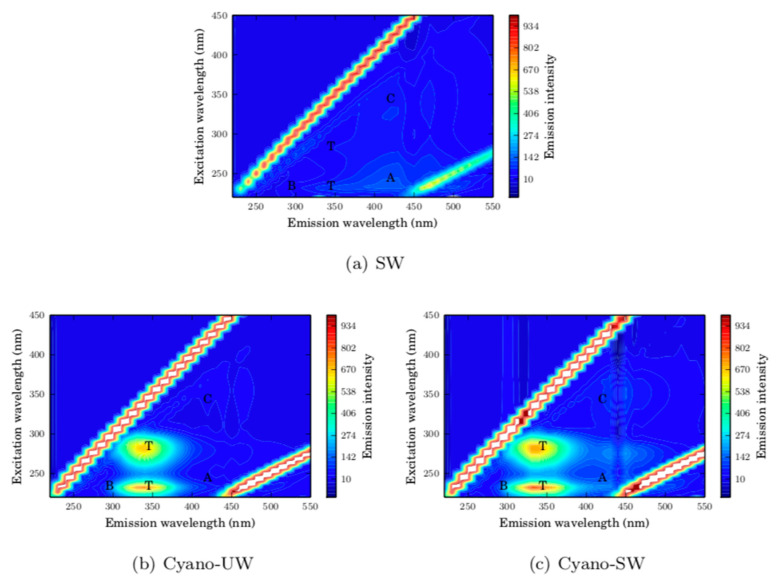
Fluorescence excitation-emission matrices of hydraulic backwash effluents. The peaks A (excitation/emission: 237-260/400-500 nm) and C (excitation/emission: 300-370/400-500 nm) represent the humic-like and fulvic-like substances. The B (excitation/emission: 225-237/309-321 and 275/310 nm) and T (excitation/emission: 225-237/340-381 and 275/340 nm) peaks represent the tyrosine protein-like and the tryptophan protein-like substances [21].

In Figure 3, the decrease in specific flux caused by this fouling is not reversed by hydraulic backwashes when the membrane is fouled by SW. In fact, given the absence of peaks in the FEEM of the SW hydraulic backwash effluent, as seen in Figure 6, suggests that the hydraulic backwash is completely ineffective. This supports the idea that natural organic matter adsorbs on the membrane surface and within the membrane pores since desorption is a thermodynamically unfavorable process and, therefore, is not removed by hydraulic forces [9].

In comparison, the hydraulic backwashes help recover the membrane’s specific flux when the membrane is fouled by cyanobacteria (Cyano-UW and Cyano-SW). This is confirmed by the FEEM of the Cyano-UW and Cyano-SW hydraulic backwash effluents, in which intense proteinic (T) peaks are observed and suggests that this recovery is mainly due to the removal of cyanobacteria cells from the membrane surface. However, although the hydraulic backwashes considerably improve the membrane specific flux for the Cyano-UW and Cyano-SW feeds, a fraction of the fouling remains irreversible as seen by the incomplete flux recovery in Figure 3. In fact, in the SW, Cyano-UW, and Cyano-SW experiments, respectively, there was a residual of 7.1, 16.4, and 22.7 µg of TOC per centimeter square of membrane surface immediately before the extended backwash (specific volume filtered: 0.4 m^3^/m^2^).

### 3.3. Ozone Chemically enhanced Backwash Kinetics

**Figure 7:**
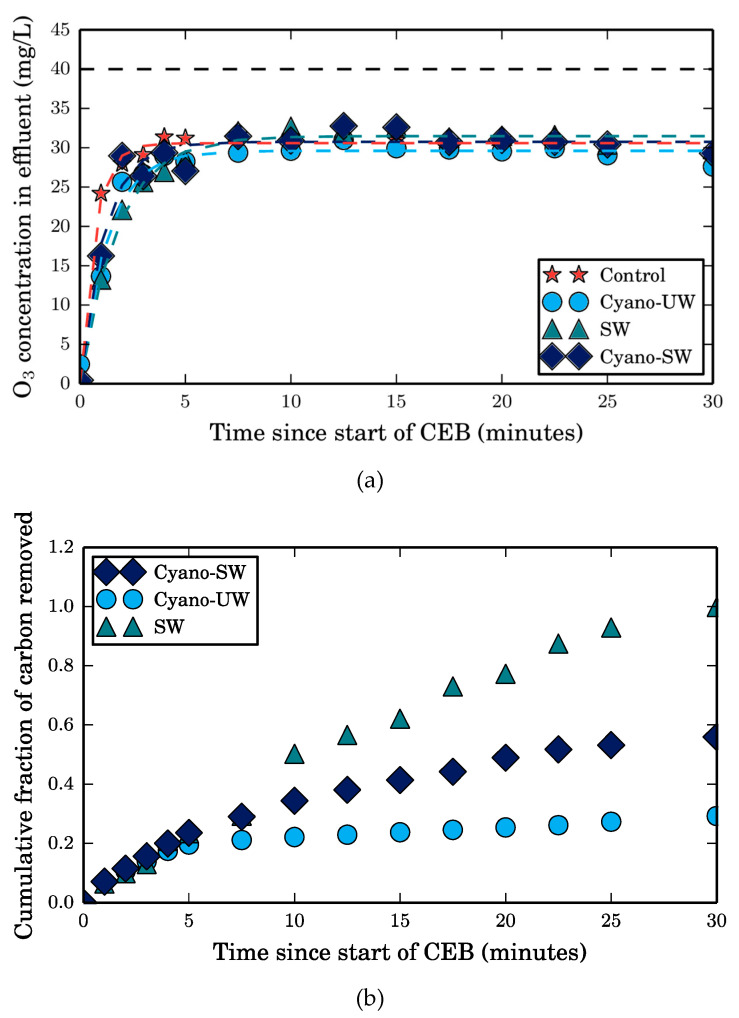
(**a**) Residual ozone concentration and (**b**) accumulated TOC in effluent at different times throughout a 30-min ozone CEB. The dissolved ozone concentration in the CEB influent was maintained at 40 mg/L, as indicated by the dashed horizontal line in Figure a. The CEB flux was approximately 600 LMH.

To control hydraulically irreversible fouling, a 30-min ozone chemically enhanced backwash was executed after 0.4 m3/m2 specific volume of feed solution filtered. The residual ozone concentration and the cumulative TOC in the effluent were monitored throughout the CEB, the results of which are presented in Figure 7.

Initially, the rate of removal of Cyano-UW foulants (cyanobacteria cells) to be similar to the removal of SW foulants in the first 5 min of the CEB. The initial rate of Cyano-SW foulants is also similar, suggesting that this initial removal is independent of the selectivity of ozone, as opposed to what was observed in the batch tests. This suggests that the removal of irreversible fouling caused by cyanobacteria is not simply due to its reactivity with ozone but due to the combination of ozone and the CEB’s hydraulic force. More precisely, ozone likely alters the foulants’ surface chemistry and, in turn, weakens the cake’s structure and increases its porosity. Therefore, it is more easily removed by hydraulic forces. The importance of the hydraulic shear might be less important in the case of SW NOM given that it is likely adsorbed in the membrane pores, as discussed in Section 3.2.

Altogether, the observations discussed above highlight the potential important impacts of CEB parameters on its performance. For instance, it could be hypothesized that a greater CEB flux could increase foulant removal. Furthermore, it could be hypothesized that complete cyanobacteria disintegration by ozone might not be required for foulant removal from the membranes. In other words, ozone doses may not need to be as large as the highest one we tested, i.e., 19 mg O_3_/mg C (equivalent ozone dose for a 30-min CEB when the membrane is fouled by Cyano-SW). A smaller dose might sufficiently weaken the cake structure for it to be removed by the CEB’s hydraulic force. The previous statement is especially true given that, after the first 7 min of the CEB, the unreacted residual ozone concentrations in the CEB effluents are relatively large, as seen in Figure 7a. Additionally, the residual ozone concentrations after the first 7 min of the CEB are the same in the SW, Cyano-UW, and Cyano-SW experiments, as well as in the control experiment (CEB on a clean membrane). Therefore, the net ozone demand (defined as the residual ozone concentration in the effluent of the SW, Cyano-UW, or Cyano-SW subtracted from the residual ozone concentration in the control’s CEB effluent), is effectively null at this point. Consequently, no additional TOC is removed in the Cyano-UW CEB. Interestingly, carbon is still removed from the membrane in the SW and Cyano-SW CEBs. Given that there is a 10 mg O_3_/L difference between the CEB influent and effluent in the control experiment (CEB of clean membrane), it is believed that ozone reacts with the membrane material to produce highly reactive hydroxyl radicals through catalytic ozonation [23]. Since this difference is also observed during the CEBs of all three feed solutions, it is suspected that catalytic ozonation could explain the continued SW foulant removal. More precisely, the adsorbed ozone and the radicals might be reacting with the SW NOM adsorbed in the membrane pores.

### 3.4. Effect of Ozone Dose on CEB Performance

**Figure 8:**
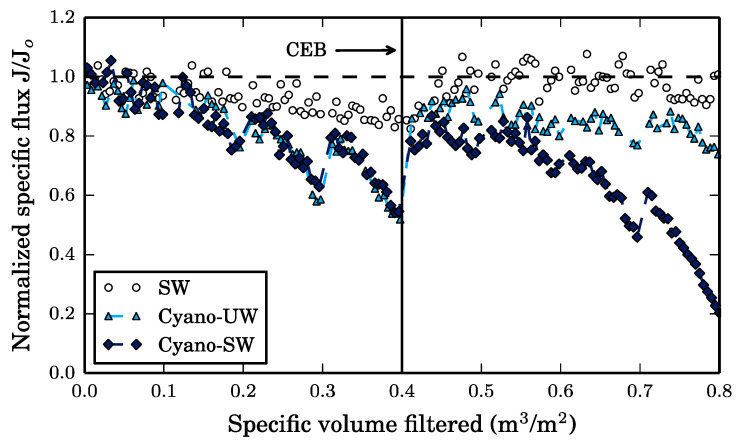
Decrease in the membrane’s specific flux during constant flux, dead-end filtration (200 LMH) of different feed solutions. The horizontal dashed line indicates the clean membrane flux. A 30-min ozone CEB was initiated after 2 h of filtration (600 LMH, 40 mg O_3_/L). Note that the Cyano-UW and the Cyano-SW are nearly aligned until the CEB.

The impact of the 30-min CEB discussed in Section 3.3 on the membrane’s specific flux is illustrated in Figure 8.

When the membrane was fouled by SW, its specific flux was fully recovered after the CEB and was even maintained during filtration afterwards. As mentioned in Section 3.3, this is likely due to the reactivity of ozone in the ceramic membrane pores, which mitigates the diffusion of SW NOM [24]. Full recovery was actually expected since the 30-min CEB completely removed the organic matter that was left on the membrane before the CEB, as seen in Figure 7b. In contrast, no organic matter is removed after 7 min of the Cyano-UW CEB, yet there is still 1.14 mg of TOC (74%) on the membrane according to the mass balance. It is suspected that leftover cell debris adsorbed on the membrane surface and in the pores could explain why only approximately 80% of the specific flux is recovered by the CEB when the membrane is fouled by Cyano-UW and Cyano-SW solutions. In fact, it was previously shown that cyanobacteria cells are not fully disintegrated by ozone [2]. This was also observed in the batch ozonation tests, in which there was a residual of 0.8 mg O_3_/mg C in the Cyano-UW solution after a 30-min exposure and in which the ozone consumption rate became almost null (see Figure 2). However, particulate organic matter (cells and associated debris) is still in solution since the TOC was not completely converted to DOC (DOC to TOC ratio only increased from 6.4% to 46%). If the cell debris are adsorbed into the membrane pores, it is probably difficultly removed by the CEB’s hydraulic shear force.

**Figure 9:**
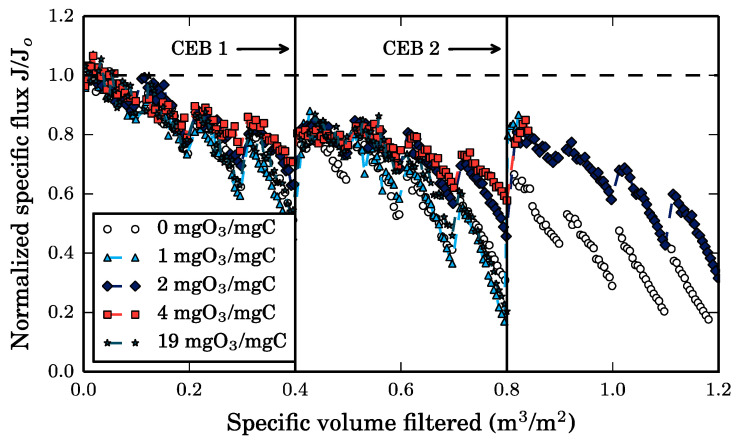
Impact of ozone CEB dosage on the membrane’s specific flux decrease during constant flux, dead-end filtration (200 LMH) of cyanobacteria-spiked surface water (Cyano-SW). The horizontal dashed line indicates the clean membrane flux. The 19 mgO_3_/mgC experiment was stopped at 0.8 m^3^/m^2^.

Nonetheless, more SW TOC is removed with CEB duration in the Cyano-SW experiment. However, the 30-min CEB is equivalent to an ozone dose of 19 mg O_3_/mg C in the Cyano-SW experiment, which is relatively large (41 mg O_3_). To assess the impact of the ozone dose on the CEB performance, the experiments were repeated with different CEB durations to obtain ozone doses of 1, 2, and 4 mg O_3_/mg C, as illustrated in Figure 9. These experiments ran over multiple CEB cycles.

**Figure 10:**
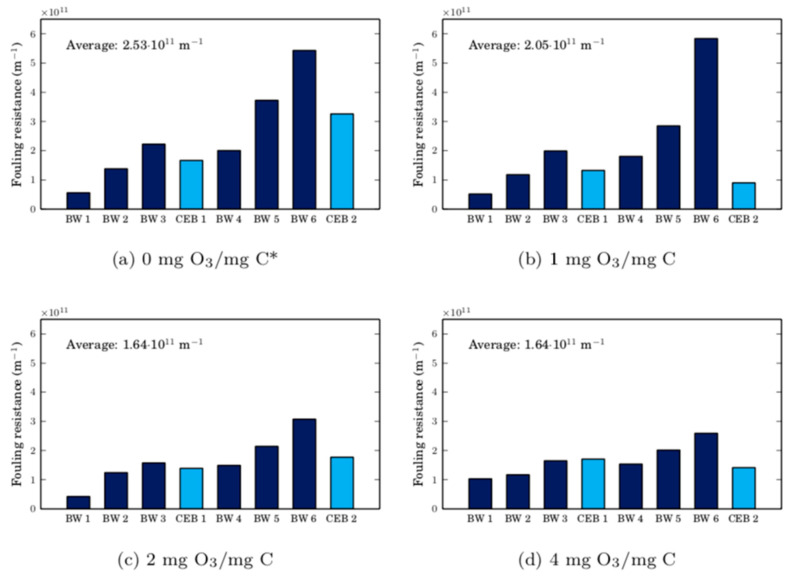
Membrane fouling resistances after hydraulic backwashes (BW) and ozone chemically enhanced backwashes (CEB) initiated during the filtration of Cyano-SW. The average fouling resistance of all cycles is provided as a metric to capture the effect of repeated CEB on long-term membrane performance. *In the baseline experiment (0 mg O_3_/mg C), the CEB was replaced with an extended hydraulic backwash.

To quantify the resistance caused by fouling throughout the experiments illustrated in Figure 9, Darcy’s law [14] was used as written in Equation (3).

(3) $$R=\frac{\Delta P}{\mu J}$$

where R is the total resistance, ΔP is the trans-membrane pressure, μ is the water viscosity, and J is the flux of water through the membrane. The intrinsic membrane resistance was calculated from the flux and pressure recorded while filtering ultrapure water through the clean membrane. On average, it was 5.63 ± 0.27 × 10^11^ m^−1^. The resistance caused by the remaining foulants after a hydraulic backwash (or a CEB) was determined by calculating the total resistance immediately after the hydraulic backwash (or the CEB) and subtracting the average intrinsic membrane resistance. The resistance caused by irreversible Cyano-SW foulants are presented in Figure 10. On average, for all ozone doses tested, the resistances before CEB 1 and CEB 2 were 1.03 ± 0.13 × 10^12^ m^−1^ and 1.91 ± 0.93 × 10^12^ m^−1^. The relatively large standard deviations for these resistances are a result of the variability of the cyanobacteria culture’s state over the study’s duration and due to the achievable accuracy of cell counts when preparing the volumes of feed required for the experiments.

As in the 30-min CEB, the membrane resistance is greater than the clean membrane resistance after an ozone chemically enhanced backwash for all tested ozone doses. After CEB 1, the remaining fouling resistances are similar in all experiments, including the baseline (0 mg O_3_/mg C) experiment. In other words, the addition of ozone in CEB 1 was only marginally advantageous. After CEB 2, the resistance is 46% smaller than in the 0 mg O_3_/mg C experiment when the ozone dose is greater than 0. It is likely that the cake layer was more compressed under the larger trans-membrane pressure required to maintain a constant permeate flux before CEB 2. Ozone can weaken the structural integrity of this compressed cake layer and increase its porosity by reacting with cells and the dissolved organic matter adsorbed onto them [14]. Additionally, the zeta potential of damaged cells is more negative than the zeta potential of live cells [5], strengthening the repulsive electrostatic forces between themselves, organic foulants, and the membrane. Presumably, the CEB’s hydraulic force can remove the weakened foulant cake layer more easily. Once again, the importance of the CEB’s hydraulic force is highlighted. It can even be concluded that when membrane fouling is dominated by a compressible cake, the role of ozone is to transform the hydraulically irreversible fouling into hydraulically reversible fouling. Given that, from a practical perspective, it would be complex to manage the waste generated by an ozone CEB, it would be important to optimize the hydraulic backwash parameters (frequency—not necessarily on a time basis, duration, flux) to control membrane fouling caused by cyanobacteria.

An observation of interest is that the resistance after CEB 2 is only slightly higher on average than the resistance after CEB 1 in experiments with ozone. It is possible that ozone chemically enhanced backwashes can keep the fouling resistance fairly constant. In other words, CEB-irreversible fouling accumulates at a very slow rate between CEBs. For economic purposes, it would be interesting to determine the smallest ozone dose required to maintain this performance. Furthermore, it should be noted that the similar resistance values observed after CEB 2 for all ozone dosages is somewhat contradictory to what might be expected when considering the TOC removal observed with the CEBs. As seen in Figure 7b, for the Cyano-SW experiment, more TOC is removed with increased CEB time, which relates to increasing ozone dose in this study. It was therefore expected that a greater organic matter removal would result in a decrease in foulant resistance, but that was not the case. As mentioned earlier, the removal of TOC after 7 min of CEB is due to NOM removal, which is believed to adsorb in the membrane pores and have less of an impact on fouling resistance than cyanobacteria cells. Interestingly, however, larger ozone doses appear to reduce the rate of irreversible fouling between CEBs, likely due to greater removal of NOM from within the membrane pores. This is captured by the average fouling resistance over all eight cycles presented in Figure 10, which initially decreases rapidly but appears to stabilize at doses above 2 mg O_3_/mg C.

## 4. Conclusions

This research demonstrates that the removal of irreversible membrane fouling caused by cyanobacteria is not simply due to the cyanobacteria cells’ reactivity with ozone, but due to the combination of ozone and the CEB’s hydraulic force. Over two filtration cycles, the average fouling resistance was reduced by 35% when ozone chemically enhanced backwashes were initiated at doses of 2 mg O_3_/mg C or higher when compared to hydraulic backwashes only. In regard to the foulant removal mechanisms involved in the ozone CEB, the following insights were gained:

1. In batch test ozonation, the components in surface water react more rapidly with ozone than with cyanobacteria. During membrane filtration, this selectivity is not observed during the initial stages of a CEB. In this case, the foulant cake is likely weakened by ozone, increasing its porosity and allowing the cake to be more easily removed by the CEB’s hydraulic force.

2. All of the surface water foulants had been removed from the membrane module. Due to the incomplete removal of cyanobacteria foulants from the membrane surface, the membrane’s specific flux recovery was not maximized. Mass balances indicated that 74% of cyanobacterial foulants were still on the membrane surface after a 30-min CEB.

## Figures and Tables

**Table 1:** Average feed water parameters.

Parameter	Units	SW	Cyano-UW	Cyano-SW
pH	-	6.90 ± 0.221	5.70 ± 0.078	7.18 ± 0.326
Turbidity	NTU	0.133 ± 0.0218	1.92 ± 0.141	2.23 ± 0.371
TOC	mg/L	2.81 ± 0.197	1.97 ± 0.318	4.58 ± 0.231
DOC	mg/L	2.81 ± 0.197	0.127 ± 0.0311	2.76 ± 0.283
UV_254_	cm^−1^	0.053 ± 0.003	0.035 ± 0.003	0.088 ± 0.001
SUVA	*L*/mg*m	1.90 ± 0.069	1.77 ± 0.117	1.93 ± 0.069

## Acronyms

SW	Surface water
Cyano-UW	Cyanobacteria-spiked ultrapure water
Cyano-SW	Cyanobacteria-spiked surface water
BW	Hydraulic (water-only) backwash
CEB	Chemically enhanced backwash
CIP	Clean-in-place
TOC	Total organic carbon
DOC	Dissolved organic carbon
NOM	Natural organic matter
